# Coenzyme Q_10_ Analogues: Benefits and Challenges for Therapeutics

**DOI:** 10.3390/antiox10020236

**Published:** 2021-02-04

**Authors:** Juan M. Suárez-Rivero, Carmen J. Pastor-Maldonado, Suleva Povea-Cabello, Mónica Álvarez-Córdoba, Irene Villalón-García, Manuel Munuera-Cabeza, Alejandra Suárez-Carrillo, Marta Talaverón-Rey, José A. Sánchez-Alcázar

**Affiliations:** Centro Andaluz de Biología del Desarrollo (CABD-CSIC-Universidad Pablo de Olavide), and Centro de Investigación Biomédica en Red: Enfermedades Raras, Instituto de Salud Carlos III, Universidad Pablo de Olavide, 41013 Sevilla, Spain; juasuariv@gmail.com (J.M.S.-R.); carmenj3b@gmail.com (C.J.P.-M.); sulevapovea@gmail.com (S.P.-C.); monikalvarez11@hotmail.com (M.Á.-C.); villalon.irene@gmail.com (I.V.-G.); mmuncab@upo.es (M.M.-C.); asuacar1@gmail.com (A.S.-C.); martatalrey@gmail.com (M.T.-R.)

**Keywords:** coenzyme Q_10_, analogues, medical applications, antioxidant, therapies

## Abstract

Coenzyme Q_10_ (CoQ_10_ or ubiquinone) is a mobile proton and electron carrier of the mitochondrial respiratory chain with antioxidant properties widely used as an antiaging health supplement and to relieve the symptoms of many pathological conditions associated with mitochondrial dysfunction. Even though the hegemony of CoQ_10_ in the context of antioxidant-based treatments is undeniable, the future primacy of this quinone is hindered by the promising features of its numerous analogues. Despite the unimpeachable performance of CoQ_10_ therapies, problems associated with their administration and intraorganismal delivery has led clinicians and scientists to search for alternative derivative molecules. Over the past few years, a wide variety of CoQ_10_ analogues with improved properties have been developed. These analogues conserve the antioxidant features of CoQ_10_ but present upgraded characteristics such as water solubility or enhanced mitochondrial accumulation. Moreover, recent studies have proven that some of these analogues might even outperform CoQ_10_ in the treatment of certain specific diseases. The aim of this review is to provide detailed information about these Coenzyme Q_10_ analogues, as well as their functionality and medical applications.

## 1. Introduction

In aerobic organisms, oxygen is an essential molecule for energy production. This process, even though fundamental for cell survival, can paradoxically be highly detrimental. Excessive cellular metabolism and oxygen consumption leads irrevocably to the production of reactive oxygen species (ROS). ROS are reactive molecules that represent the main source of cellular oxidative stress due to their ability to oxidize DNA, proteins and lipids [1]. At physiological levels, ROS are also important signaling molecules but when extreme levels of ROS are reached, the consequences can be highly disruptive to cell homeostasis [2]. Several diseases have been directly linked with ROS or the failure of its clearing mechanisms. Among them, neurodegenerative diseases [3], cancer [4], diabetes [5] or inflammatory bowel disease [6] are worth highlighting. Antioxidants, owing to their ability to counteract free radicals and neutralize oxidants, have traditionally been used to treat or palliate the symptoms of ROS-related diseases [7]. One of the best-known antioxidants is Coenzyme Q_10_ (CoQ_10_). This ubiquitous quinone acts as an electron carrier in the mitochondrial electron transport chain, mediating the transport of electrons from complex I or II to complex III. Its reduced form, ubiquinol, also acts as an antioxidant and mostly elicits its function in cell and organelle membranes [8]. Coenzyme Q_10_ has extensively been used in the medical field, ranging its therapeutic applications from neurodegenerative diseases such as multiple system atrophy (MSA) to conditions such as Barth syndrome, heart failure, fibromyalgia or insulin resistance [9]. 

In spite of the exceptional outcomes of CoQ_10_ therapies, the clinical use of this potent antioxidant is hindered by its low bioavailability [10]. The oral bioavailability of CoQ_10_ is compromised by the prominent hydrophobicity of its ten unit-long isoprenoid tail [11] as well as its instability to light and thermolability [12]. In the aim to improve the intestinal absorption of CoQ_10_, a wide variety of formulations have been developed. The majority of them have used lipid-based vehicles for the intraorganismal distribution of CoQ_10_. Liposomes, self-nanoemulsifying delivery systems (SNEDDs) and oleogels are some of these innovative formulations, which although promising, are still far from being ideal. The barrier of CoQ_10_ hydrophobicity has greatly been overcome by approaches such as CoQ_10_ micellization or its encapsulation either on lipid-free nanoparticles or β-cyclodextrin inclusion complexes [9]. 

Nevertheless, the delivery of therapeutically-effective Coenzyme Q_10_ doses remains problematic. Several studies demonstrate that due to its hydrophobicity and large molecular weight, Coenzyme Q_10_ must be administered in substantially high doses for it to reach organs like the kidneys, muscles or brain [13]. However, according to randomized human clinical trials, the daily dose of Coenzyme Q_10_ should not exceed 1200 mg, despite the fact that no severe toxic effect has been linked to the administration of larger doses of this quinone. This might be a significant limitation for the effective application of CoQ_10_- based treatments for several disorders.

This limitation was the main factor triggering the development of Coenzyme Q_10_ analogues with improved properties. These analogues were mostly created by introducing modifications in the hydrophobic tail of CoQ_10_ (Idebenone, Mitoquinone, Decylubiquinone, short-chain CoQ_10_) or by modifying the radicals of Coenzyme Q_10_’s quinone moiety (CoQ with altered C6 position). They have been the subject of study for years now and their features and their specific characteristics are generally well understood. Moreover, some of them have proved effective for the treatment of numerous diseases, ranging from neurodegenerative diseases to cardiovascular conditions or cancer. In this line, there are several ongoing clinical trials aimed to evaluate the therapeutic efficacy of certain analogues. All in all, there is currently a wide cohort of alternative molecules that might outperform CoQ_10_ in terms of ease and efficacy for medical purposes.

## 2. The Relevance of Coenzyme Q_10_

CoQ_10_ is a versatile lipophilic molecule which participates in many functions in the organism. CoQ_10_ is mainly required as a proton and electron carrier in the mitochondrial respiratory chain (MRC) [14]. In mitochondria, CoQ_10_ is also required for pyrimidine biosynthesis [15], as a lipophilic antioxidant [16], as a mitochondrial permeability transition pore (PTP) regulator [17] and for the maintenance of body temperature via its role as a cofactor for the mitochondrial uncoupling proteins [18]. Moreover, CoQ_10_ has extramitochondrial activities such as being a recycler of oxidized antioxidants like vitamin E [19], an inflammasome regulator [20] with anti-inflammatory effects [21], an autophagy modulator [22], a regulator of the physicochemical properties of cell membranes [23] and as a ferroptosis inhibitor [24]. All of these functions are possible due to its chemical structure based on a benzoquinone ring conjugated to a ten-unit long isoprenoid chain, which makes it a lipophilic/hydrophobic molecule with the ability to act as an efficient electron carrier and a free radical scavenging antioxidant in cell membranes. CoQ_10_ can transport up to two electrons, therefore it exists in three redox states: fully oxidized (ubiquinone), partially reduced (semiquinone or ubisemiquinone) and fully reduced (ubiquinol). This variable CoQ_10_ redox status plays an essential role in mitochondria, since it is as a key metabolic sensor that fine-tunes mitochondrial supercomplexes’ configuration in order to match the prevailing substrate profile [25]. Due to its extensive roles and versatility, Coenzyme Q_10_ has always been a promising therapeutic approach for the treatment of numerous diseases. Nevertheless, the quest for an improved, more efficient, more bioavailable CoQ_10_ has triggered the development of highly interesting analogue molecules that are worth looking into.

## 3. Most Relevant Coenzyme Q_10_ Analogues

### 3.1. Idebenone

Idebenone is a synthetic quinone with prominent similarities to the naturally occurring CoQ_10_. Structurally, it bears the same quinone moiety than CoQ_10_ but is characterized by the presence of a much shorter and less lipophilic tail. Strikingly, despite its analogy to CoQ_10_, Idebenone is not synthesized by any organism and cannot be isolated from any natural source [26]. It is a novel chemical compound developed in the 1980s by Takeda Pharmaceuticals as part of a medicinal chemistry approach that aimed to generate pharmacologically active entities [27]. The physicochemical properties of Idebenone mainly differ from those of CoQ_10_ due to substantial differences between their tails. The natural quinone presents a tail with 10 isoprenyl units (accounting for a total of 50 carbon atoms) with a strong hydrophobic character. Contrastingly, Idebenone’s tail is only 10 carbon atoms long and bears a terminal hydroxyl group that enables its polarity. These opposing features explain the differences between the uptake of CoQ_10_ and Idebenone. While the first is slowly absorbed from the intestinal tract (t_max_ of 6–8 h) [13], the synthetic quinone presents a much faster intestinal absorption (t_max_ of 1–2 h) [28]. Moreover, Coenzyme Q_10_ presents an elimination half-life of 33 h [13] whereas Idebenone is metabolized within minutes of its administration and therefore, no Idebenone is detectable within plasma after 1 h [29]. It has been proposed that Idebenone’s activity and efficacy resides in these sub-metabolites, as proven by Giorgio et al. [30]. Even though CoQ_10_ and Idebenone share multiple intracellular functions, there is compelling evidence to suggest that this analogue exerts new functions. In the context of mitochondrial respiration, Idebenone has been identified as an efficient substrate for complex II and III [31] and contrary to CoQ_10_, a slow substrate for complex I. Moreover, it has been demonstrated that Idebenone can inhibit the activity of complex I through the blockade of its Coenzyme Q_10_ binding pocket, hence preventing the physiological reduction of the endogenous quinone [32,33]. It would seem reasonable to believe that, given the importance of complex I, Idebenone would compromise cellular homeostasis. Nevertheless, it has extensively been demonstrated that in the presence of CoQ_10_, Idebenone activates alternative pathways to circumvent dysfunctional complex I [33,34]. The best known of these pathways is mediated by the cytoplasmic enzyme NADH-quinone oxidoreductase 1 (NQO1) [35]. This enzyme reduces Idebenone upon entering the cell as part of a response to detoxify quinones and prevent ROS production. Then, the reduced Idebenone enters mitochondria, where it is directly oxidized by complex III. By donating electrons from the cytoplasm to complex III, Idebenone successfully negotiates complex I to complex III electron transport, whose impairment would otherwise be fatal for cellular fitness [36]. 

Another of these interesting pathways is the glycerophosphate (G3PDH) shuttle, which also provides mitochondria with energy from a non-mitochondrial source [37]. Interestingly, this mechanism has mostly been observed to be active in tissues with a high energy demand. It has been reported that physiological levels of CoQ_10_ are required for Idebenone to efficiently activate this metabolic pathway. However, the mechanism through which Idebenone mediates such activation remains elusive [38]. 

The fact that Idebenone, in the presence of CoQ_10_, leads to a shift from complex I-dependent respiration to alternative pathways either involving complex II-dependent substrates or cytoplasmic substrates that are fed to complex III is of great interest given that most mitochondrial disorders are caused by complex I dysfunction. In light of this evidence, it would be reasonable to prioritize the application of Idebenone-based therapies rather than CoQ_10_ treatments on patients of complex I-related diseases such as Leigh syndrome, mitochondrial encephalomyopathy, lactic acidosis and stroke-like episodes (MELAS), Duchene Muscular Dystrophy or glaucoma [39,40]. 

Apart from its implication on the mitochondrial respiratory chain, Idebenone functions as a potent intracellular antioxidant in vitro and in vivo [41,42]. Several studies on the topic have proved the efficacy of this synthetic quinone against ROS-induced toxicity but there is still no consensus as to which working concentration of Idebenone is needed for an effective protection against oxidative stress. It is relevant to point out that in order to elicit its antioxidant or electron donor activity, Idebenone must be in its reduced hydroquinone form [43]. As previously mentioned, the cytoplasmic reductase NQO1 mediates Idebenone activation upon its entry into the cell. Unlike other quinones, Idebenone does not require the activity of mitochondrial respiratory complexes for its activation. This feature is especially convenient for the treatment of mitochondrial disorders’ patients, whose mitochondria are severely impaired. 

On top of this, the ability of Idebenone to protect complex II and III from lipid peroxidation damage is worth mentioning. It is widely known that lipid peroxidation-derived changes on the mitochondrial membrane leads to the impairment of the activity of complexes II, III and V. Recent studies have demonstrated that in this context, Idebenone treatment not only protected the function of complex III [44], with which it interacts, but also that of complex II [45]. This protective characteristic was observed in human tissue cultures, further reinforcing the clinical interest on Idebenone as a therapeutic entity.

In fact, Idebenone has been tested as a treatment for several mitochondrial diseases. The therapeutic efficacy of this CoQ_10_ analogue is especially remarkable on Leber’s hereditary optic neuropathy (LHON). This maternally inherited disease is provoked by mutations on the mitochondrial DNA (mtDNA) that lead to impairment of complex I and thus to retinal ganglion cells’ death. For its antioxidant properties, as well as its ability to act as an electron carrier, overcoming mitochondrial complex I deficiency by transferring electrons directly to mitochondrial complex III, Idebenone is the most adequate antioxidant to treat LHON patients [46]. Indeed, as reported in a recent clinical trial with a large cohort of patients, Idebenone succeeded in promoting the recovery of visual acuity in most of the patients [47]. Moreover, it was observed that the beneficial effect of Idebenone persisted despite discontinuation of the treatment [48]. 

Apart from its unparalleled therapeutical performance on LHON patients, this synthetic quinone stands out for its efficacy as a treatment for Friedreich ataxia (FRDA). From 1990 and up to the present, several clinical trials have tested the impact of Idebenone supplementation on patients of FRDA. These studies have proven that the quinone ameliorates patients’ conditions through the improvement of neurological function (reduced general weakness, improvement in fine movement and speech, and decreased difficulty in swallowing) [49] and cardiac hypertrophy (reduction in interventricular septal wall thickness, left ventricular posterior wall thickness, or left ventricular mass index) [50,51]. Idebenone has been widely tested in clinical trials for several years. Its main relevance is in FRDA [52,53,54,55], Duchenne muscular dystrophy [56,57,58] and multiple sclerosis [59,60], all of them with promising results. Currently, Idebenone has passed phase III and new applications in clinical trials are rising, such as Parkinson’s disease [61], LHON [62] or MELAS syndrome [63]. These clinical trials confirm the safety and efficacy of Idebenone, particularly when administered at higher doses [27]. 

The therapeutic spectrum of this polar CoQ_10_ analogue also comprises conditions such as pulmonary fibrosis [64], dementia [65], MELAS [66] and glaucoma [67]. 

All in all, in addition to the functions it shares with CoQ_10_ (antioxidant capacity and the ability to donate electrons to complex III), Idebenone presents an impressive repertoire of features of great medical interest. For this reason, it should be regarded as a promising alternative, or even improvement, to conventional CoQ_10_ therapies. 

### 3.2. Mitoquinone

Mitoquinone, also named MitoQ, is another of the more widespread CoQ_10_ analogues [68]. This mitochondrial-targeted antioxidant was developed in the 1990s by covalently attaching ubiquinone or CoQ_10_ to the lipophilic decyltriphenylphosphonium (dTPP) cation through a 10-carbon aliphatic chain [69]. The most relevant feature that differentiates MitoQ from other CoQ_10_ analogues is its ability to selectively concentrate on the mitochondrial membrane. This remarkable capacity is owed to the dTPP cation, which crosses the mitochondrial lipid bilayer and accumulates several-hundred fold in the mitochondrial matrix driven by the large potential of the organelle’s inner membrane [70]. Once in the matrix, the ubiquinone moiety of MitoQ is reduced by complex II yielding ubiquinol, which acts as a potent antioxidant [71,72]. This ubiquinol moiety is constantly recycled to the active antioxidant by the respiratory chain. Moreover, the precise localization of MitoQ in the matrix-facing surface of the inner membrane favors the ability of this molecule to protect the components of the mitochondrial electron transport chain (ETC) from lipid peroxidation [73]. Even though it has been observed that Mitoquinone mainly reacts with lipid peroxidation products, further research is required to define its exact mode of action [74]. Another interesting asset of MitoQ is its fast uptake from the circulation into cells following oral or intravenous (IV) administration [75]. This enhanced bioavailability with respect to CoQ_10_ has boosted the clinical application of the synthetic quinone, which has become one of the most recurrent alternatives to traditional CoQ_10_ therapies. 

Even though MitoQ was initially conceived to protect the mitochondrial membrane from lipid peroxidation [73], it is currently used to treat a broad range of conditions. For instance, studies have demonstrated that MitoQ not only restores the mitochondrial membrane potential in heart failure induced by pressure overload but also mitochondrial respiration and calcium retention capacity. On top of this, 14-week treatment with MitoQ reduced the ROS over-production associated with this syndrome [76]. Still in the field of cardiovascular diseases (CVD), MitoQ has been tested as a therapy for hypertension in humans. According to the authors of this study, 6-week supplementation with MitoQ improved vascular endothelial function, reduced aortic stiffness and decreased oxidized LDL (a circulating marker of oxidative stress) in middle-aged or older adults with a hypertension background. In light of these observations, the authors claim that MitoQ may be an effective treatment for improving vascular function and thus decreasing the risk of CVD [77]. 

However, it is important to point out that the applications of this CoQ_10_ analogue go way beyond vascular conditions. In fact, MitoQ has been found to be extremely effective for the treatment of diabetic kidney disease (DKD). The kidney protective function of MitoQ has been linked to (1) its ability to restore mitophagy via Nrf2-mediated regulation of PINK transcription; (2) its reduction of mitochondrial oxidative stress; and (3) its capacity to ameliorate aberrant mitochondrial dynamics, which would otherwise trigger tubular injury and apoptosis attenuation under high glucose conditions [78]. 

Interestingly, apart from preserving the kidneys from damage, MitoQ has been reported to protect the liver through several mechanisms. In alcoholic fatty liver disease MitoQ has been observed to successfully prevent ethanol-induced oxidant-damage and liver steatosis through a mechanism involving ROS/reactive nitrogen species (RNS) scavenging and the suppression of Hypoxia-inducible factor 1-alpha (HIF1α) activation [79]. Furthermore, according to the authors of a recent clinical trial, MitoQ decreased necroinflammation in the liver of chronic Hepatitis C patients, as indicated by the significant reduction of alanine transaminase (ALT) and aspartate aminotransferase (AST) reported in their blood’s plasma [80].

Neurodegeneration is undoubtedly one of the medical fields at which MitoQ has been most extensively studied due to its promising therapeutic potential. In vitro studies have proven that MitoQ protects neuronal models of Parkinson’s disease (PD) against 1-methyl-4-phenyl-1,2,3,6-tetrahydropyridine (MPTP)-induced behavioral deficit, tyrosine hydroxylase (TH)-positive neuronal loss, depletion of striatal dopamine, inactivation of mitochondrial aconitase, neuronal apoptosis and cell death [81]. Additionally, application of MitoQ on a PD-zebrafish model increased mitochondrial function and improved antioxidant balance as well as neurotransmitter levels in the fish [82]. This evidence suggests that MitoQ could be a highly effective treatment for Parkinson’s disease patients. This mitochondria-targeted quinone also seems to be of great interest for therapeutic applications on Alzheimer’s disease (AD). Indeed, MitoQ-attenuated β-amyloid (Aβ)-induced neurotoxicity in cortical neurons and prevented increased production of reactive species and loss of mitochondrial membrane potential in them [83]. Moreover, exposure to MitoQ increased lifespan and promoted the healthspan of transgenic Aβ-overexpressing C. elegans nematodes [84]. On top of this, the synthetic antioxidant prevented cognitive decline as well as oxidative stress, Aβ accumulation, astrogliosis, synaptic loss, and caspase activation in a mouse model of AD [83]. All in all, it would seem reasonable to support the use of MitoQ as a therapy for diseases involving oxidative stress and metabolic failure, like AD. As a matter of fact, there is compelling evidence to suggest that MitoQ-based treatments would be highly beneficial for Huntington’s disease [85], amyotrophic lateral sclerosis [86] and traumatic brain injury [87] patients. 

There are countless clinical trials on humans related to mitoquinone: Parkinson disease [88], multiple sclerosis [89,90], metabolic dysfunction in asthma [91], aging [92], hepatitis C [93] and non-alcoholic fatty liver disease [94]. Currently, some of them are still ongoing. Interestingly, the efficacy of this CoQ10 analogue is variable, being successful for the treatment of certain conditions but totally ineffective in others. 

MitoQ is unquestionably a promising antioxidant with a wide variety of potential clinical applications. However, the molecular mechanisms through which it might elicit its therapeutic functions are still not completely understood. Further research is required to fully comprehend the versatility of this quinone and to possibly unravel some of its still undisclosed features.

### 3.3. Decylubiquinone

Decylubiquinone (DUb), 2,3-dimethoxy-5-methyl-6-decyl-1,4-benzoquinone, is a synthetic CoQ_10_ analogue at which the isoprenoid side chain of CoQ_10_ is substituted by a saturated decyl hydrocarbon chain that favors its passive localization to the mitochondrial membranes [95]. This modification is critically involved in the antioxidant or pro-oxidant properties of the ubiquinone analogue [96]. Like CoQ_10_, DUb is able to take electrons from complex I to be reduced into decylubiquinol, which subsequently transfers electrons to complex III. Hano et al. [97] showed the effect of DUb on the steady state kinetics of complex I in bovine heart mitochondria concluding that the binding of DUb induced a conformational change in the shape of the binding site, which allows the binding of a quinone with a long isoprenoid side chain. DUb may be used favorably as an alternative to CoQ_10_ because the interaction of DUb with complex I is more similar to that between endogenous ubiquinone and complex I [98].

Jayne E. et al. [99] studied the effects of DUb on the activities of mitochondrial complexes in rat brain synaptosomes. They concluded that DUb can enhance the activities of supercomplexes such as I/III and II/III but is not determinant in the activities of the individual complexes, I, III, and IV, or in the rate of oxygen consumption, which was completely unaffected by DUb. The precise mechanism by which DUb increases the activities of supercomplexes [100] remains unknown. Jayne E. et al. propose that the addition of decylubiquinone may increase the rate of electron transfer from complex I to complex III, resulting in increased complex I/III-specific activities. Although there is little evidence on the involvement of complex II in supercomplexes, a study has suggested that complex II can associate with complexes III and IV or with complexes I, III, and IV to form supercomplexes [101]. In fibroblasts derived from Leber Hereditary Optic Neuropathy (LHON) harboring the m.11778G > A mutation, DUb highly decreases reactive oxygen species (ROS) from affected and control cells [102].

Although DUb is known for being a CoQ_10_ analogue and presumably keeps its beneficial effects, it also shows new effects as a mitochondrial PTP modulator [96]. In cancer research, the combination treatment of DUb, an X-linked inhibitor of the anti-apoptotic protein (XIAP) and EDL-360 significantly inhibited glioma growth by inducing apoptosis, which shows that DUb has anticancer activity [103]. Furthermore, combination treatment of DUb with thialysine significantly suppressed the viability of human acute leukemia Jurkat T cells [104]. DUb is also capable of inhibiting breast cancer growth and metastasis by suppressing tumor-induced angiogenesis. DUb suppresses angiogenesis via the ROS/p53/BAI1 signaling pathway in vascular endothelial cells [105]. The findings indicate that DUb could exert an important effect on cancer progression and treatments associated with the inhibition of angiogenesis.

Keeping in mind both effects, antioxidant and PTP modulators, Murad et al. [106] showed an attenuation on the levels of systolic blood pressure, LDL-cholesterol and malondialdehyde, and an increase in HDL cholesterol levels in stroke-prone spontaneously hypertensive (SHRSP) in a rat model. As a result, DUb can also be considered a new potential antihypertensive, hypolipidemic and antioxidant therapeutic agent on the prevention and treatment of diseases linked to oxidative stress. Currently, there are no ongoings clinical trials despite decylubiquinone’s presumed potential.

### 3.4. Plastoquinone and SKQ1

There are plenty of known antioxidants capable of removing ROS from mitochondria; however, most of them can also act as prooxidants depending on the dosage and the circumstances [107,108]. For this reason, Antonenko et al. [109] started the search for a therapeutically useful, rechargeable antioxidant operating without risk of prooxidant side effects. Plastoquinone was their starting molecule. This ubiquinone analogue operates in the chloroplast electron transfer chain as an antioxidant, while the electrons in the MRC are served by ubiquinone. The presence of an exclusive quinone for ROS removal in the chloroplast emphasizes the potent antioxidant effect of plastoquinone [110]. The presence of this chloroplast-exclusive ubiquinone remarks the elevated oxidative stress present in the chloroplast [111]. This high oxidative stress present in the chloroplast is mainly caused by the high light absorbance by chlorophyll [112] and the acidic environment of the thylakoid [113], both elements enhance the ROS production in the cell.

To make plastoquinone able to penetrate mitochondria, Antonenko et al. synthesized several plastoquinone derivatives combined with various penetrating ions and tested them in model membranes, mitochondria, cells and organisms. These compounds were named after Skulachev Ion and Quinone (SkQ) [114]. Cationic plastoquinone derivatives penetrate planar phospholipid membrane [115], only accumulate in mitochondria [116], and can be reduced by complex III in the MRC [109]. Nevertheless, the antioxidant mechanisms of SkQ have not been described in full detail. For instance, the exact mechanism through which SkQ1 interferes with the superoxide-producing sites of the MRC or other sources of ROS remains elusive. Two principal hypotheses have been proposed to explain the antioxidant ability of SkQ1 [117]. One of them is based on the fatty acid co-mediated uncoupling and has a strong support in bioenergetics [118], the other suggests an ion-pairing mechanism for administered drugs and endogenous compounds [119].

In isolated mitochondria, among all synthetized SkQs, SkQ1 was found to operate as a very potent antioxidant at lower concentrations protecting cardiolipin against oxidation by OH^-^. Although SkQ1 showed strong prooxidant effects at higher concentrations, the threshold between antioxidant and prooxidant concentrations for SkQ1 is as wide as about 1000, whereas for its ubiquinone-containing analogue MitoQ it was less than 2 under the same conditions [120]. In cell cultures, SkQ1 inhibited H_2_O_2_-induced apoptosis and mitochondrial fragmentation, and stimulated mitochondrial fusion [121].

Nowadays, SkQ1 is being used in many fields: inflammation [122,123], wound healing [122,124], tumor growth suppression [125], Alzheimer’s disease [126], fertility [127], aging [128,129], immunoregulation [130], ischemia [131] and mitochondrial diseases [132,133] among others. All of them are related with mitochondrial function and ROS homeostasis. It is important to highlight the importance of SkQ1 as an anti-aging molecule or geroprotector since it has been reported to increase lifespan of several animal models from crustaceans up to mice [134,135]. SkQ1 acts mainly by decelerating the age-related decay of the immune system as an involution of thymus and spleen follicles and a reduction of the ratio of lymphocytes to neutrophils in blood [116]. However, the effect on the lifespan is only visible in non-sterile vivariums. In mice, the effect of SkQ1 is also accompanied by significantly slowing down most age-related processes such as osteoporosis, sarcopenia or loss of vision [135,136].

There are several clinical trials using SkQ1 to treat dry-eye syndrome [137,138,139]. This pathology affects around 20% of global population, with a higher incidence in older people [140]. Dry-eye syndrome disease is a chronic condition of the corneal surface marked by persistent symptoms of irritation or burning that can cause inflammatory damage to the cornea and conjunctiva if untreated. Although it is considered a multifactorial disease, one study reached phase 3, indicating that SkQ1 is safe and efficacious for the treatment of dry eye signs and symptoms [141].

### 3.5. C6 Position 

In the CoQ_10_ molecule, the antioxidant activity is believed to originate at the quinone nucleus, which is the reason why keeping the quinone nucleus and introducing some hydrophilic groups at the C6 position could increase its antioxidant effects and bioavailability. In order to better understand the structure reactivity relationship of CoQ_10_ analogues as antioxidants and to find some potential therapeutic agents for oxidative stress-related diseases, Wang et al. [142] synthesized a series of 2,3-dimethoxy-5-methyl-1,4- benzoquinones substituted at the C6 position with various methoxy-, hydroxyl- and heterocyclic groups, and looked for their antioxidant effects against 2,2-diphenyl-1- picrylhydrazyl (DPPH) in vitro. They also established a protocol for the synthetic synthesis of several novel CoQ_10_ analogues by C6 substitution. Among CoQ_10_ and its analogues tested, those containing piperazine and morpholine at the C6 position of CoQ_10_ exhibited higher antioxidant activities than those containing hydroxyalkyl or alkoxy-substituents at the same position. Their best antioxidant was C6 N-benzoylpiperazine CoQ_10_ that showed better radical scavenging activities than standard CoQ_10_. Furthermore, C6 piperazine CoQ_10_ was highly soluble in water, meaning that this compound would have a more potent antioxidant activity than CoQ_10_ in hydrophilic environments.

These results confirm that the rational design of CoQ_10_ analogues as novel antioxidants is possible and efficient. These CoQ_10_ analogues are just the tip of the iceberg for the development of potential therapeutic antioxidants to treat oxidative stress-related diseases. Unluckily, Wang et al. did not follow their research on these compounds.

### 3.6. Short Chain Coenzyme Q_10_

Apart from Idebenone, many kinds of CoQ_10_ analogues with distinct lengths of the isoprenoid side-chain lengths exist in nature. In humans and most higher organisms, CoQ_10_ is the only quinone acting at the ETC [143]; however rodents possess CoQ_9_ as the main quinone analogue together with a small amount of CoQ_10_ [144]. Aerobic bacteria such as *Escherichia coli* and yeast as *Saccharomyces cerevisiae* have CoQ_8_ and CoQ_6_ as their main analogues respectively [143]. On the other hand, CoQ with shorter isoprenoid side chains than CoQ_5_ are barely observed as main quinones in organisms. Nevertheless, they have been frequently used as intermediaries during in vitro respiratory chain enzyme studies rather than endogenous CoQ because of their higher water solubility.

Short chain quinones have been studied as therapeutic molecules due to their ability to reduce oxidative stress [145], enhance mitochondrial electron transfer [34], and modulate apoptosis [146]. Kagan et al. [147] revealed a correlation between the isoprenoid side chain length and antioxidant potential of the CoQ_10_ analogues, in which the antioxidant efficiency of the CoQ_10_ analogues increases as the length of their chain shortens. However, it has been reported that some CoQ with a shorter isoprenoid side chain than CoQ_4_ could be cytotoxic to cultured mouse embryonic myocardial cells [148] and induce the apoptosis of the human leukemia B-cell line BALL-1 [149]. Moreover, there is evidence that the shortest chain CoQ_10_ analogues, such as CoQ_2_ or CoQ_3_, are inhibitors of complex I [150] and promoters of ROS production in the MRC [151], since they are complex I binding site competitors and are very inefficient in the NADH electron transfer process [152]. In contrast, there are no reports about the toxicity of long length CoQ such as CoQ_11_, CoQ_12_, or CoQ_13_.

The main quinone cellular metabolism pathway requires two consecutives steps: First NADPH:cytochrome P450 reductases generate semiquinones by incomplete, one-electron reduction [153]. However, these semiquinones are mostly unstable and prone to generate ROS [154]. Then NQOs, as seen in the Idebenone section, complete the two-electron reduction of quinones and their derivates [154]. The whole process synthetizes hydroquinones, also known as quinols, without the risk of ROS release. Furthermore, the relevance of NQOs is based on their capacity to mitigate the noxious effect of several toxins and prooxidants [155]. For instance, NQOs have been shown to be involved in the reduction of several drugs such as quinone epoxides, aromatic nitro and nitroso compounds, azo dyes and Cr(VI) compounds [156], with NQO1 showing its highest specificity towards quinones. With respect to benzoquinones, NQOs are able to efficiently reduce CoQ_0_ [157] and CoQ_1_ [145]. Erb M et al. [34] described that effectiveness of short chain quinones in restoring the mitochondrial function as strongly being dictated by the hydrophilicity/lipophilicity of the entire molecule rather than particular structural features. These parameters determine the reduction by NQO1, the influence levels of lipid peroxides by their antioxidant function and finally govern their interaction with the ETC.

In order to find novel treatment for mitochondria diseases, Chan T et al. [145] used CoQ_1_ and a rotenone-cellular-based model to study the molecular mechanism of cell death by complex I inhibition. They found that CoQ_1_ was able to prevent rotenone-induced hepatocyte cytotoxicity but also restore ATP levels, mitochondrial membrane potential and respiration. This observation suggested that the CoQ_1_ could reactivate the ETC after severe mitochondrial damage. The CoQ_1_ cytoprotection may result from its reduction by NQO1 using NADH to form Q_1_H_2_. This reoxidation of NADH may restore cellular redox potential and prevents cytotoxicity. They also noted that Q_1_H_2_, when formed, could act as an electron bypass and reduce complex III to promote ATP formation, however this effect was not correlative with higher concentrations, in contrast to the antioxidant capacity. In addition, the superoxide radicals formed by complex I may be scavenged by Q_1_H_2_. Overall, the cytoprotective effect of CoQ_1_ was attributed to its ability to act as an electron acceptor and/or an antioxidant rather than by acting as an electron bypass to restore ATP. No cytoprotection occurred with NQO1-inactivated hepatocytes. 

Erb M et al. [34] performed a large quinone screening with 70 related quinone compounds including idebenone, decylubiquinone and several short-chain quinones. These compounds were tested for their ability to rescue ATP levels in three different cell lines: human myoblasts 9Te, rat myoblast L2 and immortalized human hepatocytes HepG2. They showed that structural modifications to the side chains determining the physicochemical properties of the molecules were influencing their ATP rescue activity, rather than modifications in the quinone moiety itself. Although previous studies had remarked that the ATP rescue activity is dependent on reduction by NQO1 in cells [36], no clear correlation between the reduction by NQO1 in a cell-free assay system and cellular ATP rescue activity was found. Some compounds successfully activated NQO1, however they failed to protect against rotenone-induced loss of ATP. On the other hand, some compounds showed significant ATP-rescue activity in cells but hardly any reduction by NQO1 in cell free assays. This discrepancy suggested that other parameters in addition to reduction by NQO1 also influence the ability to rescue ATP levels under conditions of impaired complex I. Depending on the hydrophilicity/lipophilicity balance, prooxidant tendency and dosage, they concluded that just a few short-chain quinones could be beneficial for ROS/mitochondrial impairment-related diseases.

Opposed to the classic antioxidant perspective of short chain quinones, Takahashi T et al. [146] focused on their ability to induce apoptosis. They observed different toxicities of each quinone chain length against HeLa cells and they stablished the following rank of potency: CoQ_0_ >> CoQ_3_ ≈ CoQ_1_ ≥ CoQ_2_ >> CoQ_4_. CoQ_0_ was the most toxic compound to HeLa cells among the compounds tested. The study showed that the reduction of the short chain of CoQ by NQO1 could induce apoptosis of HeLa cells by ROS and p53 in an independent manner; however, the exact mechanism supporting this process is still unknown.

Interestingly, a recent study has proven that CoQ_4_, at lower doses than those used by Takahashi et al., functions as Coenzyme Q_10_ in the mitochondrial respiratory chain of patients with ubiquinone deficiency [158]. This implies that CoQ_4_ could be a promising therapeutic alternative to CoQ_10._ To further reinforce this, it has been observed that dietary supplementation with CoQ_4_ succeeded to rescue a *Drosophila* model of Coenzyme Q_10_ deficiency [159].

### 3.7. EPI-743/Vatiquinone

EPI-743 or vatiquinone is a *para*-benzoquinone analog resulting from the combination of both CoQ_10_ and vitamin E molecules that presents improved pharmacologic properties and therapeutic efficacy than its constituents alone. In vitro, EPI-743 has been shown to be approximately one thousand- to ten thousand-fold more potent than CoQ_10_ or idebenone in protecting mitochondria during extreme prooxidant environments [160]. EPI-743 is considered safe, orally absorbed and crosses the blood–brain barrier [160]. Its biological activity depends on the intrinsic properties of the *para*-benzoquinone moiety to undergo a reversible two electron cycling reaction [160]. This molecule, also known as α-tocotrienol quinone, exerts its effect on the activity modulation of oxidoreductases, in particular NQO1, resulting in increased cellular glutathione (GSH) concentration and improvement of the REDOX status. In addition, EPI-743 may be able to regulate the gene expression profile of antioxidant mechanisms, including GSH biosynthesis [161]. GSH plays a key role in the cellular free radical defense and is mainly distributed across the cytoplasm [162]. GSH also acts on various organelles, including peroxisomes, the nuclear matrix, endoplasmic reticulum and mitochondria [163]. Recently, Kahn-Kirby A. et al. remarked the relevance of EPI-743 demonstrating that it is able to regulate the balance between glutathione peroxidase 4 (GPX4) and 15- lipoxygenase 15 (15-LO) [164]. The loss of this equilibrium promotes the activation of the ferroptosis pathway, which is a form of iron- and lipid-dependent regulated cell death associated with GSH depletion and production of lipid peroxides by lipoxygenase enzymes [165]. The ferroptosis process is directly linked to several disorders such as epilepsy [166], mitochondrial pathologies [167,168], cancer [169] and neurodegeneration [170].

EPI-743 has been successfully tested in various clinical trials, principally focused on mitochondrial diseases [171]. In one study with LHON patients [172], EPI-743 treatment arrested disease progression and reversed vision loss in all but one of five treated patients. In LHON patients, EPI-743 counters disease progression and improves quality of life by increasing the GSH cellular pool [173,174]. In Leigh syndrome patients, administration of EPI-743 reduced hospitalization and adverse events of the pathology [175,176,177]. Furthermore, EPI-743 has passed phase 2 trials in Parkinson’s disease [178], Rett syndrome [179] and Pearson’s syndrome [180]. Currently, EPI-743 is gaining relevance for the treatment of epilepsy [164,166] and Friedreich ataxia, being responsible for a significant improvement in neurological function and an arrest of disease progression [167,181,182]. 

Taking together all the positive results, EPI-743 is presented as a promising molecule for many mitochondrial-related diseases. However, most of the clinical assays rely on a small number of patients due to the rarity of these pathologies. Despite an equal administration of EPI-743, there is a high variability in response between patients due to individual differences in drug absorption and metabolization [161]. In addition, some authors noted that EPI-743’s co-administration with other palliative drugs can alter in some way its final activity [183].

## 4. Conclusions

Overall, the beneficial effects of CoQ_10_ on human health and disease treatment are well known. However, there is growing interest among the scientific community for CoQ_10_ analogues and their presumably optimized performance in antioxidant therapies. In this review we have outlined the chemical improvements that successfully enhance CoQ_10_ bioavailability: shortening of its isoprenoid chain (idebenone and short chain CoQ analogues); addition of specific radicals to promote its mitochondrial accumulation (mitoquinone); modification of natural analogs to boost their antioxidant effect (plastoquinone); modification of the quinone ring (C6 modifications); and introduction of changes on its isoprenoid chain (decylubiquinone) to diversify its biology, its hybridization with other antioxidants and to enhance its potency (EPI-743). Taken together, these synthetic CoQ_10_ analogues open the door to new and improved therapies for conditions ranging from mitochondrial diseases to cancer. 

**Figure 1:**
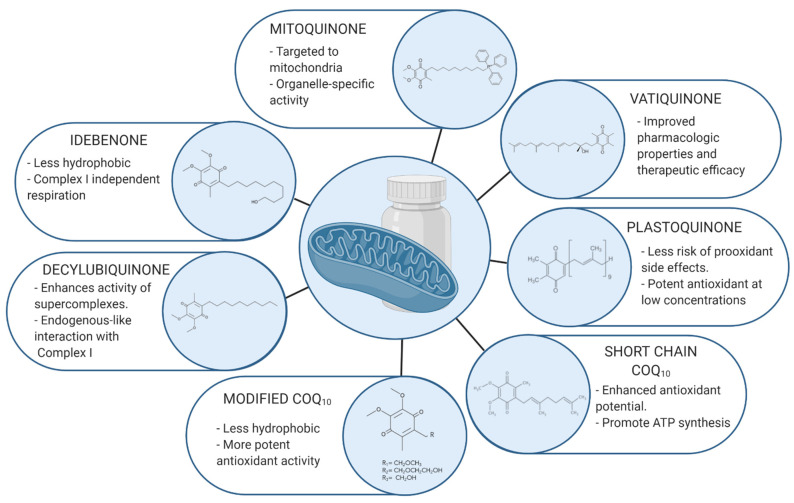
Molecular representation of CoQ_10_ analogues. The quinone ring is known to exert the main antioxidant effect of the molecule, whereas the isoprenoid chain is able to modify the lipophilicity/hydrophilicity ratio. By altering or adding new radicals to both structures we are able to develop new treatments in order to improve specificity and functional properties.

All the reviewed analogues are summarized in Table 1 and Figure 1.

## Figures and Tables

**Table 1:** Coenzyme Q_10_ analogues and their applications.

Coenzyme Q_10_ Analogue	Medical Applications	Level of Study	References
Idebenone	LHON	Approved for patients’ treatment	[46,47,48]
	Friedreich ataxia	Patients’ treatment	[27,49,50,51]
	Pulmonary fibrosis	Tested in vivo	[64]
	Dementia	Patients’ treatment	[65]
	MELAS	Patients’ treatment	[66]
	Glaucoma	Patients’ treatment	[67]
Mitoquinone	Heart failure	Tested in vivo	[76]
	Hypertension	Tested in vivo	[77]
	Diabetic kidney disease	Tested in vivo	[78]
	Alcoholic fatty liver disease	Tested in vivo	[79]
	Hepatitis C	Patients’ treatment	[80]
	Parkinson’s disease	Patients’ treatment	[81,82]
	Alzheimer’s disease	Tested in vivo	[83,84]
	Huntington’s disease	Tested in vivo	[85]
	Amyotrophic lateral sclerosis	Tested in vivo	[86]
	Traumatic brain injury	Tested in vivo	[87]
Decylubiquinone	LHON	In vitro studies	[102]
	Cancer	Tested in vivo	[103,104,105]
	Hypertension	Tested in vivo	[106]
SkQ1	Inflammation	Tested in vivo	[122,123]
	Wound healing	Tested in vivo	[122,124]
	Tumor growth suppression	In vitro studies	[125]
	Alzheimer’s disease	Tested in vivo	[126]
	Fertility	Tested in vivo	[127]
	Aging	Tested in vivo	[128,129]
	Immunoregulation	Tested in vivo	[130]
	Ischemia	Tested in vivo	[131]
	Dry eye treatment	Patients’ treatment	[140,141]
CoQ_10_ with modifications on C6	ROS related diseases	-	-
Short-Chain CoQ_10_	CoenzymeQ10 deficiency-related syndromes	Tested in vivo	[145]
	Apoptosis modulation	In vitro studies	[146]
EPI-743	LHON	Patients’ treatment	[172]
	Leigh syndrome	Patients’ treatment	[175,177,184]
	Parkinson’s disease	Patients’ treatment	[178]
	Rett syndrome	Patients’ treatment	[185]
	Pearson’s syndrome	Patients’ treatment	[180]
	Epilepsy	In vitro studies	[164]
	Friedreich’s ataxia	Patients’ treatment	[167,181,182]

## Abbreviations

ROS	Reactive Oxygen Species
CoQ_10_	Coenzyme Q10
MRC	Mitochondrial respiratory chain
MSA	Multiple system atrophy
NQO1	Cytoplasmic enzyme NADH-quinone oxidoreductase 1
G3PDH	Glycerophosphate
MELAS	Mitochondrial encephalomyopathy, lactic acidosis and stroke-like episodes
LHON	Leber’s hereditary optic neuropathy
mtDNA	Mitochondrial DNA
FRDA	Friedreich Ataxia
dTPP	Decyltriphenylphosphonium
ETC	Electron transport chain
IV	Intravenous
CVD	Cardiovascular disease
DKD	Diabetic Kidney Disease
RNS	Reactive Nitrogen Species
HIF1α	Hypoxia-inducible factor 1-alpha
ALT	Alanine transaminase
AST	Aspartate aminotransferase
MPTP	1-methyl-4-phenyl-1,2,3,6-tetrahydropyridine
TH	Tyrosine hydroxylase
PD	Parkinson Disease
AD	Alzheimer Disease
Aβ	β-amyloid
